# Assessment of the Function of Respiratory Muscles in Patients after COVID-19 Infection and Respiratory Rehabilitation

**DOI:** 10.3390/tropicalmed8010057

**Published:** 2023-01-12

**Authors:** Anna Romaszko-Wojtowicz, Michał Szalecki, Karolina Olech, Anna Doboszyńska

**Affiliations:** 1 Department of Pulmonology, School of Public Health, Collegium Medicum, University of Warmia and Mazury in Olsztyn, 10-719 Olsztyn, Poland; 2 The Centre for Pulmonary Diseases in Olsztyn, 10-357 Olsztyn, Poland; 3 Central Clinical Hospital of the Ministry of Interior and Administration in Warsaw, 02-507 Warsaw, Poland

**Keywords:** MIP, MEP, COVID-19, rehabilitation, respiratory muscles function

## Abstract

Objectives: The MIP (maximum inspiratory pressure) and MEP (maximum expiratory pressure) are sensitive indicators of respiratory muscle function. The aim of the study was to assess the function of respiratory muscles in patients after COVID-19 infection, before and after hospitalisation at the Pulmonary Rehabilitation Ward. Materials and Methods: The study was conducted on a group of 19 people with laboratory-confirmed COVID-19 infection, who, in the period from 1 February to 31 May 2021, were hospitalised at the Independent Public Pulmonary Hospital and underwent respiratory rehabilitation in hospital conditions. A statistical analysis was performed using the STATISTICA package, ver. 10. A respiratory pressure meter (RP Check) was used to measure muscle strength. Measurements were performed twice on each patient—before admission and after hospitalisation in the Pulmonary Rehabilitation Ward. Results: We show that conducting pulmonary rehabilitation contributes to the increase in MIP and MEP, which are associated with increased strength of the inspiratory and expiratory muscles. The average value of MIP increased by 11.95 cmH_2_O and MEP by 26.16 cmH_2_O. The improvement was visible in both female and male patients. Conclusions: Pulmonary rehabilitation contributes to the improvement of respiratory muscle function indicators among patients after COVID-19 infection. Assessment of the MIP and MEP indices is a simple and quick way to reliably assess the function of the respiratory muscles.

## 1. Introduction

Viral infections, including COVID-19 (coronavirus disease 2019) caused by the SARS-CoV-2 virus, can lead to lung damage, possibly to acute respiratory deficiency syndrome (ARDS) and, as a further consequence, to respiratory failure that requires invasive mechanical ventilation. Lung tissue damage leads to physical activity limitations and shortness of breath, which is associated with impaired gas exchange. However, it is believed that it may also be related to the limitation of respiratory muscle strength [1]. The reasons for this phenomenon are various. Avoiding activities that cause shortness of breath leads to impaired muscle function. Inflammation of the interstitial tissue of the lungs can also affect the muscles [2]. Not only that, the treatment often used in COVID-19—corticosteroids—can also have an impact. They may cause myopathy [3,4]. In this study, we decided to attempt an objective assessment of respiratory muscle strength based on the analysis of the maximum inspiratory pressure (MIP) and maximum expiratory pressure (MEP) indices. Respiratory rehabilitation contributes to the improvement in patients’ clinical conditions [5]. However, it can be difficult to “measure” this improvement. For this purpose, we decided to use a device that is a digital respiratory pressure meter (manometer). It is a simple and cheap tool, useful in everyday clinical practice, enabling a non-invasive assessment of respiratory muscle strength. The maximal inspiratory pressure and maximal expiratory pressure are indicators of the strength of inspiratory and expiratory muscles, respectively [6]. An increase in the strength of inspiratory muscles is responsible for generating under-pressure in the pleural cavity and a resulting increase in lung capacity [7]. The degree of the chest expansion not only depends on the work of external intercostal muscles affecting the transverse dimension of the chest but is also linked to the work of the diaphragm in the sagittal plane. An increase in expiratory volume affects an increase in the expiratory volume. Weakened inspiratory or expiratory muscles affect the volume of the lungs. The MIP (maximal inspiratory pressure) and MEP (maximal expiratory pressure) are sensitive indicators of the respiratory muscle functions [6]. When the strength of respiratory muscles weakens, even healthy ones begin to limit their physical effort, while those with cardiac insufficiency are more likely to do so [8,9]. Kaneko et al., who studied 51 men with chronic obstructive pulmonary disease (COPD), showed that a limitation of the function of the lungs was associated with a decreased mobility of the chest wall [10]. Measurement of the respiratory muscles’ strength is a tool used to assess the function of the muscles. It is also used in the evaluation of patients with neuromuscular diseases and in patients with lower respiratory tract infections [11,12].

The purpose of this study was to assess the function of respiratory muscles in patients after COVID-19 infection, prior to and after hospitalisation at a rehabilitation ward.

## 2. Materials and Methods

The research participants were interviewed to obtain basic demographic and medical data. The participation in the study was completely voluntary and anonymous. Each participant had the right to refuse to provide any answer and to withdraw from the study without giving any reason. The study was carried out during hospitalisation, without processing personal data. Patients with a laboratory-confirmed (PCR test) diagnosis of COVID-19 were included in the study. Mild COVID-19 patients were considered as patients with mild symptoms without pneumonia, moderate as patients with non-severe pneumonia, and severe as patients who needed hospitalisation in the ICU [13]. Contraindications to participate in the study were: aneurysms, uncontrolled hypertension, and urinary incontinence [14]. Before the examination, each patient was informed about the research procedure and was later given the results together with recommendations when it was completed. The study was approved of by the Bioethics Committee at the University of Warmia and Mazury in Olsztyn, Collegium Medicum, Faculty of Medicine, No. 18/2021.

### 2.1. Measurement of Muscular Strength

A digital respiratory pressure meter (manometer) (RP Check) was used to measure the strength of the muscles [15,16,17,18]. An RP Check is a device that enables a non-invasive measurement of the strength of respiratory muscles by recording the MIP, MEP, MRR (maximal rate of relaxation), and MRPD (maximal rate of pressure development). The MRPD is assessed according to the initial slope of the maximal respiratory pressure curve reflecting the function of the muscles.

### 2.2. The Course of an Examination

Measurements were performed twice on each patient—first before admission and second after hospitalisation in a pulmonary rehabilitation ward. The rehabilitation was planned by qualified medical personnel (pulmonologists and rehabilitation specialists). All rehabilitation activities were conducted under the supervision of nurses and physiotherapists. Measurements of the respiratory function were carried out by a properly trained physiotherapist. Before measurements, each patient made 5 test recordings of their maximal inhalations and exhalations in order to become familiar with the procedure. The maximal expiratory pressure was measured at the maximal respiratory effort maintained for at least one second. The best MEP values from three acceptable and repeatable trials—in the range of 10% differences—were recorded. The values thus obtained were expressed in absolute values (cmH_2_O).

### 2.3. Statistical Description

The statistical analysis was supported by the STATISTICA, ver. 10 software package (StatSoft Inc., www.statsoft.com (accessed on 10 September 2022)). The minimal and maximal values, standard deviation, 95% confidence interval for the mean, and median for each value were calculated. The W Shapiro–Wilk test was applied to test the normality of the distribution. The homogeneity of the variance in the groups was analysed with the Levene’s test. Any result was deemed to be statistically significant at *p* < 0.05. The Student’s *t*-test was employed when the variables satisfied the assumptions of the parametric tests. When the variables did not satisfy the assumptions of normal distribution or equality of variance, then the Mann–Whitney *U* or Kruskal–Wallis tests were used, respectively.

## 3. Results

The persons qualified for the study were patients who had COVID-19 confirmed by a laboratory test. Nineteen patients (six women and thirteen men) were included in the study. These patients were hospitalised from 1 February 2021 to 31 May 2021 in the Rehabilitation Ward of the Independent Public Pulmonary Hospital in Olsztyn. The average age of the patients was 53.32 years (SD: 8.49). The specific demographic data of the research participants are given in Table 1.

The mean duration of the patients’ hospitalisation was 13.47 days (SD: 4.10). Most—that is, 12 patients (63.16%)—needed hospitalisation before due to contracting pneumonia during COVID-19 (moderate COVID-19 cases). Two patients (10.52%) had stayed in an ICU (severe COVID-19 cases). Seven patients (36.84%) had not required hospitalisation or had not gone to hospital despite having a doctor’s referral (mild COVID-19 cases).

The mean time lapse since the positive test for COVID-19 to hospitalisation at the Rehabilitation Ward was 98.59 days (SD: 34.57).

Concurrent chronic diseases in the patients are presented in Table 2.

None of the patients were diagnosed as presenting abnormalities in gasometry during their stay in the Pulmonary Rehabilitation Ward. None of the patients were active smokers, while eight persons (42.10%) used to smoke.

It was demonstrated that the function of the respiratory muscles (MEP) improved (*p* = 0.002) following the rehabilitation in both the entire group and in the subgroups of men (*p* = 0.005) and women (*p* = 0.004) (Table 3).

## 4. Discussion

Patients after COVID-19 present secondary worsening of the lung functions and physical performance [19,20]. The strength of the respiratory muscles decreases, which entails an insufficient function of the lungs [21]. It has been demonstrated that physical training of the respiratory muscles contributes to the patient’s reduced fatigue and the feeling of exertion. Huqang et al., in a study published in Lancet, showed that over 76% of patients, about half a year after COVID-19 infection, complain of such persistent symptoms as tiredness or muscle weakness, while over 50% still show changes in imaging tests of the chest [22]. Carfi et al. also proved that, 60 days after recovering from COVID-19 infection, 87.4% of patients still complain of at least one symptom, most often shortness of breath or fatigue [23].

Particular benefits from respiratory rehabilitation are gained by persons who needed to be hospitalised in an Intensive Care Unit in the course of a COVID-19 infection due to increasing respiratory deficiency. Stam et al. demonstrated that an earlier onset of rehabilitation contributes to a shorter duration of hospitalisation and required respiratory support. They recommend starting rehabilitation as soon as possible, best during hospitalisation in an Intensive Care Unit and later continued in a specialised hospital ward [24]. However, Kiekenz et al. suggested that early respiratory rehabilitation could contribute to more severe desaturation [25]. Taking into account the situation in the health care system during the COVID-19 pandemic, McNeary et al. demonstrated that rehabilitation should begin as soon as possible after hospitalisation due to COVID-19 infection [26]. The average time elapsing from a positive test for SARS-CoV-2 to admission to a rehabilitation ward determined in our study was 3 months.

In our study, we were able to show that patients who had recovered from COVID-19 and subsequently underwent a rehabilitation treatment had a significantly better function of the respiratory muscles (MEP) than achieved prior to the rehabilitation. An increase in the MIP and MEP values, indicating that the improved strength of the inspiratory and expiratory muscles, is associated with greater mobility of the chest wall [27]. Padkao et al. proved in their research that both the MIP and MEP were significantly correlated with the expansion of the thorax and movements of the diaphragm [28]. The MEP values considered to be normal are expiratory pressure over 100 cmH_2_O for women and 130 cmH_2_O for men [29]. Moreover, it is assumed that the absolute value MEP < 80 cmH_2_O is always abnormal, indicating a systemic neuromuscular disease and predicting an ineffective cough [30]. Among the patients who participated in our study, there were eight persons (42.11%) who presented a decreased value of MEP < 80 cm H_2_O in the first test (before a rehabilitation treatment). In the subsequent test, conducted at the end of hospitalisation in the Rehabilitation Ward, this number was halved (4 out of 19 patients, i.e., 21.05% of all patients showed decreased MEP). Table 3 aggregates the values of the pressure generated before and after the implemented respiratory rehabilitation in patients who had had COVID-19.

Exercising respiratory muscles improves their strength, respiratory exercise capacity, and the thickness of the diaphragm muscle, as well as contributing to the reduction of perceived dyspnoea [31,32]. Patients with weaker respiratory muscles tend to benefit from exercising respiratory muscles. Langer et al., in their study, showed that respiratory muscle exercise was beneficial for both patients with respiratory disease and healthy persons [33]. It has also been demonstrated that the pre-operation exercise of respiratory muscles reduces the risk of post-operative respiratory complications [34]. Nikoletou et al. proved that a four-week series of respiratory exercise reduces the length of a hospitalisation period, mortality rate, and the risk of intubation in patients at risk of prolonged hospitalisation [35]. The available evidence suggests that the weakness of respiratory muscles is linked to breathlessness. Cases of the reduced function of respiratory muscles are relatively rare in the general population. However, in patients with several diseases, the risk of weakening respiratory muscles can increase or potentially intensify the severity of the decreased efficiency of respiratory muscles associated with ageing [36]. In our study, among the persons who needed to be hospitalised in the Rehabilitation Ward, 13 persons (68.42%) had a positive history of hypertension, and 3 of these persons (15.79%) had type 2 diabetes. Data concerning chronic diseases are collated in Table 2.

Despite this research and its preliminary results, the role of exercising respiratory muscles in the alleviation of SARS-CoV-2 infection respiratory complications has not yet been investigated thoroughly. However, in our opinion, and based on our research results, it can be ascertained that exercising respiratory muscles can be a successful method for limiting long-term complications. This issue nevertheless requires further investigations on larger groups of patients.

## 5. Limitations

Obviously, the study discussed in this paper has certain limitations. First and foremost, it was carried out in one hospital only, on patients hospitalised in the Rehabilitation Ward at the Independent Public Pulmonary Hospital in Olsztyn, Poland. Nineteen patients were included in the study. In order to achieve full representativeness, this group should be larger, so that it is possible to compare patients in groups, as detailed in the Section 3, according to the classification of COVID-19 severity (mild, moderate, and severe). Furthermore, due to the small group of respondents (as the sample is less than 25), it was not possible to perform all statistical tests (e.g., a regression analysis).These limitations stem purely from system-related capacities. Yet, it is worth noting that the study results unquestionably show that patients who have undergone respiratory rehabilitation after COVID-19 gain significant benefits. The presented test results were obtained during the standard hospitalisation of patients after COVID-19 infection at the rehabilitation ward. The study did not include a complete assessment of the lungs function (6-min walking test, spirometry, plethysmography, and carbon monoxide diffusion) due to the fact that not all hospitalised patients performed such tests. Unfortunately, this work does also not include quality-of-life tests; normally, they are not performed during hospitalisation. Undoubtedly it would be a worthy supplement to this study. Moreover, this is one of few published studies concerning the evaluation of the strength of muscles as a predictive factor in the assessment of the function of the lungs, especially in the mentioned group of patients. Both the American Thoracic Society (ATS) and the European Respiratory Society (ERS) recommend using spirometry as part of the screening tests in patients with dyspnoea [37]. However, performing spirometry in patients who have had COVID-19 and have been submitted for mechanical ventilation is burdened with some risk. The literature provides numerous reports and descriptions of cases of patients with iatrogenic pneumothorax and mediastinal pneumothorax, which occurred after spirometry [38,39]. The examination we propose, where an RP Check manometer is used, does not pose such a threat.

## 6. Conclusions

Patients after COVID-19 (mild, moderate, and severe) infection gain substantial benefits from respiratory rehabilitation. Pulmonary rehabilitation contributes to the improvement of respiratory muscle function. In addition, it may also contribute to the improvement of lung function. Further research is required.

## Figures and Tables

**Table 1:** Data of the patients hospitalised in the Rehabilitation Ward after COVID-19.

Value	All (*n* = 19)	Men (*n* = 13)	Women (*n* = 6)
Age (years)	53.32 (SD: 8.49)	52.67 (SD: 8.25)	54.56 (SD: 7.75)
BMI (kg/m^2^)	30.70 (SD: 4.39)	30.24 (SD: 4.04)	31.18 (SD: 4.62)
Duration of rehabilitation (days)	13.47 (SD: 4.10)	13.56 (SD: 4.09)	13.31 (SD: 3.87)

**Table 2:** Specification of the chronic diseases in the patients.

Disease	All (*n* = 19)	Men (*n* = 13)	Women (*n* = 6)
Hypertension	13 (68.42%)	10 (76.92%)	3 (50.00%)
Diabetes mellitus	3 (15.79%)	2 (15.38%)	1 (16.67%)
Embolism	2 (10.53%)	2 (15.38%)	0
Cardiovascular diseases	1 (5.26%)	1 (7.69%)	0
Pulmonary diseases	1 (5.26%)	0	1 (16.67%)
Neoplastic diseases	1 (5.26%)	1 (7.69%)	0

**Table 3:** Comparison of the strength of the respiratory muscles before and after hospitalisation in the Rehabilitation Ward.

**Expiratory:**	**MEP I (cmH_2_O)**	**MEP II (cmH_2_O)**	***p* (<0.05)**
All	97.16 (SD: 46.19)	123.32 (SD: 44.80)	0.002
Women	67.17 (SD: 42.34)	87.33 (SD: 37.48)	0.004
Men	111.00 (SD: 41.05)	139.92 (SD: 37.57)	0.005
**Inspiratory:**	**MIP I (cmH_2_O)**	**MIP II (cmH_2_O)**	***p*** **(<0.05)**
All	81.21 (SD: 30.45)	93.16 (SD: 30.33)	0.047
Women	78.88 (SD: 32.54)	87.63 (SD: 30.29)	0.086
Men	81.44 (SD: 31.26)	94.06 (SD: 34.02)	0.047

## Data Availability

The data presented in this study are available on request from the corresponding author for research purposes only.

## References

[1] Walterspacher S., Schlager D., Walker D.J., Müller-Quernheim J., Windisch W., Kabitz H.-J. (2013). Respiratory muscle function in interstitial lung disease. Eur. Respir. J..

[2] Holland A.E. (2010). Exercise limitation in interstitial lung disease-mechanisms, significance and therapeutic options. Chron. Respir. Dis..

[3] Dekhuijzen P., Decramer M. (1992). Steroid-induced myopathy and its significance to respiratory disease: A known disease rediscovered. Eur. Respir. J..

[4] Surmachevska N., Tiwari V. (2022). Corticosteroid Induced Myopathy. StatPearls [Internet].

[5] Connolly B., Salisbury L., O’Neill B., Geneen L., Douiri A., Grocott M.P., Hart N., Walsh T.S., Blackwood B., Group E. (2015). Exercise rehabilitation following intensive care unit discharge for recovery from critical illness. Cochrane Database Syst. Rev..

[6] Schoser B., Fong E., Geberhiwot T., Hughes D., Kissel J.T., Madathil S.C., Orlikowski D., Polkey M.I., Roberts M., Tiddens H.A. (2017). Maximum inspiratory pressure as a clinically meaningful trial endpoint for neuromuscular diseases: A comprehensive review of the literature. Orphanet J. Rare Dis..

[7] Lutfi M.F. (2017). The physiological basis and clinical significance of lung volume measurements. Multidiscip. Respir. Med..

[8] Janssens L., Brumagne S., McConnell A.K., Raymaekers J., Goossens N., Gayan-Ramirez G., Hermans G., Troosters T. (2013). The assessment of inspiratory muscle fatigue in healthy individuals: A systematic review. Respir. Med..

[9] Lin S.-J., McElfresh J., Hall B., Bloom R., Farrell K. (2012). Inspiratory muscle training in patients with heart failure: A systematic review. Cardiopulm. Phys. Ther. J..

[10] Kaneko H., Shiranita S., Horie J., Hayashi S. (2016). Reduced chest and abdominal wall mobility and their relationship to lung function, respiratory muscle strength, and exercise tolerance in subjects with COPD. Respir. Care.

[11] Boswell-Ruys C.L., Lewis C.R.H., Wijeysuriya N.S., McBain R.A., Lee B.B., McKenzie D.K., Gandevia S.C., Butler J.E. (2020). Impact of respiratory muscle training on respiratory muscle strength, respiratory function and quality of life in individuals with tetraplegia: A randomised clinical trial. Thorax.

[12] Rodriguez-Nunez I., Torres G., Luarte-Martinez S., Manterola C., Zenteno D. (2020). Respiratory Muscle Impairment Evaluated with Mep/Mip Ratio in Children and Adolescents with Chronic Respiratory Disease. Rev. Paul. Pediatr..

[13] Motoc N.S., Ruta V.-M., Man M.A., Ungur R.A., Ciortea V.M., Irsay L., Nicola A., Valean D., Usatiuc L.O., Matei I.R. (2022). Factors Associated with Prolonged RT-PCR SARS-CoV-2 Positive Testing in Patients with Mild and Moderate Forms of COVID-19: A Retrospective Study. Medicina.

[14] Troosters T., Gosselink R., Decramer M. (2005). Respiratory muscle assessment. Eur. Respir. Monogr..

[15] Society A.T. (2002). ATS/ERS Statement on respiratory muscle testing. Am. J. Respir. Crit. Care Med..

[16] TI D.S., FIT TING J.-W. (1999). Sniff nasal inspiratory pressure: Reference values in Caucasian children. Am. J. Respir. Crit. Care Med..

[17] Uldry C., Fitting J.-W. (1995). Maximal values of sniff nasal inspiratory pressure in healthy subjects. Thorax.

[18] Wilson S., Cooke N., Edwards R., Spiro S. (1984). Predicted normal values for maximal respiratory pressures in caucasian adults and children. Thorax.

[19] Frohlich L.F., Vieira P.J., Teixeira P.J., Silva F.A., Ribeiro J.P., Berton D.C. (2014). Exercise capacity in adolescent and adult patients with post infectious bronchiolitis obliterans. Pediatr. Pulmonol..

[20] Mattiello R., Sarria E.E., Stein R., Fischer G.B., Mocelin H.T., Barreto S.S., Lima J.A., Brandenburg D. (2008). Functional capacity assessment in children and adolescents with post-infectious bronchiolitis obliterans. J. Pediatr. (Rio J.).

[21] Meyer F.J., Borst M.M., Zugck C., Kirschke A., Schellberg D., Kubler W., Haass M. (2001). Respiratory muscle dysfunction in congestive heart failure: Clinical correlation and prognostic significance. Circulation.

[22] Huang C., Huang L., Wang Y., Li X., Ren L., Gu X., Kang L., Guo L., Liu M., Zhou X. (2021). 6-month consequences of COVID-19 in patients discharged from hospital: A cohort study. Lancet.

[23] Carfi A., Bernabei R., Landi F., Gemelli Against C.-P.-A.C.S.G. (2020). Persistent Symptoms in Patients After Acute COVID-19. JAMA.

[24] Stam H.J., Stucki G., Bickenbach J. (2020). COVID-19 and post intensive care syndrome: A call for action. J. Rehabil. Med..

[25] Kiekens C., Boldrini P., Andreoli A., Avesani R., Gamna F., Grandi M., Lombardi F., Lusuardi M., Molteni F., Perboni A. (2020). Rehabilitation and respiratory management in the acute and early post-acute phase. “Instant paper from the field” on rehabilitation answers to the COVID-19 emergency. Eur. J. Phys. Rehabil. Med..

[26] McNeary L., Maltser S., Verduzco-Gutierrez M. (2020). Navigating Coronavirus Disease 2019 (COVID-19) in Physiatry: A CAN Report for Inpatient Rehabilitation Facilities. PM R.

[27] de Cordoba Lanza F., de Camargo A.A., Archija L.R.F., Selman J.P.R., Malaguti C., Dal Corso S. (2013). Chest wall mobility is related to respiratory muscle strength and lung volumes in healthy subjects. Respir. Care.

[28] Padkao T., Boonla O., Padkao T., Boonla O. (2020). Relationships between respiratory muscle strength, chest wall expansion, and functional capacity in healthy nonsmokers. J. Exerc. Rehabil..

[29] Spiro S.G., Silvestri G.A., Agustí A. (2012). Clinical Respiratory Medicine: Expert Consult-Online and Print.

[30] Ruppel G.L., Enright P.L. (2012). Pulmonary function testing. Respir. Care.

[31] Souza H., Rocha T., Pessoa M., Rattes C., Brandão D., Fregonezi G., Campos S., Aliverti A., Dornelas A. (2014). Effects of inspiratory muscle training in elderly women on respiratory muscle strength, diaphragm thickness and mobility. J. Gerontol. Ser. A Biomed. Sci. Med. Sci..

[32] Pazzianotto-Forti E.M., Mori T., Zerbetto R., Baruki S., Montebello M.I., Pacheco E., Mendes N., Tanaka T., Reid D. (2019). Effects of inspiratory muscle training on respiratory muscle strength, physical fitness and dyspnea in obese women. Eur. Respir. Soc..

[33] Langer D., Charususin N., Jacome C., Hoffman M., McConnell A., Decramer M., Gosselink R. (2015). Efficacy of a Novel Method for Inspiratory Muscle Training in People With Chronic Obstructive Pulmonary Disease. Phys. Ther..

[34] Nepomuceno B.R.V., Barreto M.S., Almeida N.C., Guerreiro C.F., Xavier-Souza E., Neto M.G. (2017). Safety and efficacy of inspiratory muscle training for preventing adverse outcomes in patients at risk of prolonged hospitalisation. Trials.

[35] Nikoletou D., Man W.D., Mustfa N., Moore J., Rafferty G., Grant R.L., Johnson L., Moxham J. (2016). Evaluation of the effectiveness of a home-based inspiratory muscle training programme in patients with chronic obstructive pulmonary disease using multiple inspiratory muscle tests. Disabil. Rehabil..

[36] Pereira F.D., Batista W.O., Fuly P.d.S.C., Alves Junior E.d.D., Silva E.B.d. (2014). Physical activity and respiratory muscle strength in elderly: A systematic review. Fisioter. Em Mov..

[37] Qaseem A., Wilt T.J., Weinberger S.E., Hanania N.A., Criner G., van der Molen T., Marciniuk D.D., Denberg T., Schunemann H., Wedzicha W. (2011). Diagnosis and management of stable chronic obstructive pulmonary disease: A clinical practice guideline update from the American College of Physicians, American College of Chest Physicians, American Thoracic Society, and European Respiratory Society. Ann. Intern. Med..

[38] Chen X., Zhang G., Tang Y., Peng Z., Pan H. (2020). The coronavirus diseases 2019 (COVID-19) pneumonia with spontaneous pneumothorax: A case report. BMC Infect. Dis..

[39] Wong K., Kim D.H., Iakovou A., Khanijo S., Tsegaye A., Hahn S., Narasimhan M., Zaidi G. (2020). Pneumothorax in COVID-19 Acute Respiratory Distress Syndrome: Case Series. Cureus.

